# Muscle synergies and spinal maps are sensitive to the asymmetry induced by a unilateral stroke

**DOI:** 10.1186/s12984-015-0031-7

**Published:** 2015-04-18

**Authors:** Martina Coscia, Vito Monaco, Chiara Martelloni, Bruno Rossi, Carmelo Chisari, Silvestro Micera

**Affiliations:** Translational Neural Engineering Laboratory, Center for Neuroprosthetics and School of Engineering, École Polytechnique Fédérale de Lausanne, BM 3210, Station 17, 1015 Lausanne, Switzerland; Neural Engineering Area, The BioRobotics Institute, Scuola Superiore Sant’Anna, Pisa, Italy; Neurorehabilitation Unit, Ospedale Cisanello, Pisa, Italy; Bertarelli Foundation Chair in Translational Neuroengineering, Center for Neuroprosthetics and Institute of Bioengineering, School of Engineering, École Polytechnique Fédérale de Lausanne (EPFL), Lausanne, Switzerland

**Keywords:** Lower limb muscle synergies, Spinal maps in stroke subjects, Gait asymmetry after stroke

## Abstract

**Background:**

Previous studies have shown that a cerebrovascular accident disrupts the coordinated control of leg muscles during locomotion inducing asymmetric gait patterns. However, the ability of muscle synergies and spinal maps to reflect the redistribution of the workload between legs after the trauma has not been investigated so far.

**Methods:**

To investigate this issue, twelve post-stroke and ten healthy participants were asked to walk on a treadmill at controlled speeds (0.5, 0.7, 0.9, 1.1 km/h), while the EMG activity of twelve leg muscles was recorded on both legs. The synergies underlying muscle activation and the estimated motoneuronal activity in the lumbosacral enlargement (L2-S2) were computed and compared between groups.

**Results:**

Results showed that muscle synergies in the unaffected limb were significantly more comparable to those of the healthy control group than the ones in the affected side. Spinal maps were dissimilar between the affected and unaffected sides highlighting a significant shift of the foci of the activity toward the upper levels of the spinal cord in the unaffected leg.

**Conclusions:**

Muscle synergies and spinal maps reflect the asymmetry as a motor deficit after stroke. However, further investigations are required to support or reject the hypothesis that the altered muscular organization highlighted by muscle synergies and spinal maps may be due to the concomitant contribution of the altered information coming from the upper part of the CNS, as resulting from the stroke, and to the abnormal sensory feedback due to the neuromuscular adaptation of the patients.

## Background

Literature has widely documented that the activity of many muscles while achieving both upper and lower limbs related motor tasks is characterized by a modular organization [1-7]. This strategy is often described by “muscle synergies” (also named “motor modules”) representing sets of muscles outlined in weight coefficients, which are synchronously activated by basic “temporal components” (also named “activation patterns” [5,8,9] or “primitive signals” [1]; see *Materials and Methods* for further details).

The modular organization underlying muscle activity in human locomotion has been extensively investigated leading to the conclusion that the linear combination of few (between 3 and 5) basic activation patterns can generate the activity of a large amount (from 8 to 32) of muscles [1,10-13]. Simulation studies have further corroborated this result confirming that a simple neural control strategy involving five activation modules is sufficient to produce well-coordinated walking [14-16]. On the whole, these evidences suggest that muscle synergies describe the elementary biomechanical subtasks of locomotion (e.g., weight acceptance, body support and forward progression, leg deceleration) and reflect the relationship between the central control and the output of the motor tasks [2,17-19].

Due to a unilateral cerebrovascular accident, the motor control is remarkably altered resulting in an asymmetric activation of muscles during the gait cycle [20-23]. Despite of this, previous studies dealing with the effects of supraspinal impairments on muscle coordination did not unequivocally clarify the effect of the trauma on the re-organization of the modular activity underlying EMG signals between affected and unaffected sides [8,24-29]. More in detail, Clark and colleagues [24] observed that a cerebrovascular accident mainly affects the synergic spatio-temporal architecture underlying muscle activity of the affected side in chronic post-stroke patients. The altered control strategy would hence consists in merging different modules (i.e., the number of muscle synergies decreases due to the trauma and its severity) involving a reduced complexity of the motor coordination of the impaired side. Differently, Gizzi and colleagues [26] noticed that the number of modules required to describe muscle activity during walking does not change early after a stroke (i.e., subacute phase) and is similar to that of healthy subjects. Moreover, they observed that the basic temporal components underlying muscle activity appear preserved despite the trauma, even if the weight coefficients of muscle synergies are different between the two sides and with respect to those found in the healthy control group. Accordingly, the authors hypothesized that descending signals may be misdirected due to the trauma and/or the consequent CNS reorganization, and can enable different subsets of muscles [26].

The differences between previous studies were ascribed to the different cohorts of enrolled patients, since post-stroke adaptations in motor control occur more extensively in chronic patients than in subacute ones [26].

A similar hypothesis has been tested in upper limb related motor tasks of post-stroke survivors. In particular, some authors observed that synergies related to upper arm and shoulder muscles are similar between affected and unaffected sides, despite size and location of the cerebral lesion, and time elapsed from the onset of the accident [8]. Others noticed that muscle synergies could be altered due to the trauma, even if they did not reported univocal description of the alteration [27,28]. Finally, almost all authors agree on the evidence that a cerebrovascular accident affects the temporal components [28-30], disregarding results reported for locomotion [26].

Recently, the spatiotemporal activity of motoneuronal (MN) pools belonging to the spinal cord, also named “spinal map”, has been introduced as a possible tool to describe how the central pattern generator (CPG) output is directed to the MN pools within each spinal segment (see *Material and Methods* for further details) [31-33]. Spinal maps represent the spatiotemporal organization of the EMG signals and they are strongly correlated to muscle synergies [13,32,34], but appear more sensitive than muscle synergies to describe both extrinsic (e.g., speed, weight bearing) and intrinsic (e.g., spinal cord injury, ageing) constraints modifying gait patterns [13,18,35-38].

According to these evidences, muscle synergies and spinal maps seem to be able to describe different aspects of motor control from EMG signals. Specifically: muscle synergies would reflect common structures underlying the modularity of locomotion; spinal maps would reveal the re-organization of MN activity due to internal or external constraints. The investigation of spinal activity may offer insights on the origin of the asymmetry after stroke. As matter of fact, it is well know that locomotion is controlled by several structures of the CNS, and that spinal circuitries play a fundamental role for sensory motor integration and muscle pattern generation during walking [39,40].

This study was designed to investigate, for the first time, the relationship between gait asymmetry resulting after a unilateral cerebrovascular accident and the modifications of muscle synergies and spinal maps. It was hence aimed at clarifying whether the post-stroke asymmetric locomotion can be related to the unbalanced muscle recruitment within motor modules between the two limbs, as described by muscle synergies, and/or to the altered spatio-temporal organization of output of the CPG, as described by spinal maps. In this respect, we hypothesized that a unilateral cerebrovascular accident can involve a significant reorganization of the activity of spinal circuits related to the affected side. As a consequence, it will alter both muscle coordination and the spinal activity of the paretic side.

## Methods

### Participants

Twelve post-stroke patients with a single cerebrovascular accident and consequent hemiparesis were enrolled in accordance with the following requirements: (1) hemiparesis due to a single unilateral stroke and resulting in a sensorimotor disturbance of only one side; (2) no evidence of severe cognitive or language dysfunctions that would have interfered with the ability to understand instructions; (3) no evidence of neglect; (4) at the time of testing, all subjects had already carried out standard physical therapy, that consisted in occupational therapy or physiotherapy, including motor exercises, mobilization, activities of daily living, or any combination of the three; (5) ability to walk without aids during the experimental sessions, (6) absence of other significant non-stroke-related impairments. The Fugl-Meyer motor assessment scale [41] and the Hauser Ambulation Index [42] were also used for the clinical assessment. Table 1 summarizes age, anthropometric features and clinical picture for patients.

**Table 1:** **Description of patients**

**ID**	**Gender**	**Age**	**Weight [kg]**	**Height [cm]**	**Affected side**	**Months since stroke**	**Stroke type**	**Fügl-Meyer Scale** ^**1**^	**Hauser Ambulation Index** ^**2**^
P1	M	58	75	170	L	49	Ischemic	132	4
P2	M	33	75	170	L	48	Ischemic	147	2
P3	M	60	92	187	L	39	Ischemic	141	4
P4	M	65	87	165	L	63	Ischemic	216	3
P5	M	63	86	178	L	36	Ischemic	124	4
P6	M	69	72	173	R	40	Ischemic	160	4
P7	F	78	55	150	R	2	Ischemic	205	2
P8	F	71	72	166	R	60	Hemorrhagic	160	3
P9	M	48	90	183	L	27	Hemorrhagic	125	4
P10	M	61	72	178	R	8	Hemorrhagic	160	2
P11	F	73	58	155	R	61	Hemorrhagic	127	5
P12	M	23	68	180	L	222	Hemorrhagic	206	2

^1^The maximum score of the Fügl-Meyer Scale is 222 and means normal function.

^2^The Hauser Ambulation Index ranges from 0 (asymptomatic) to 9 (restricted to wheelchair, unable to self-transfer independently).

Ten healthy participants were recruited to serve as gender, age, height, and weight comparable controls for the hemiparetic patients. Healthy controls did not show any evidence or known history of postural, skeletal or neurological diseases, and exhibited normal joint range of motion and muscle strength.

Written informed consent was obtained from all subjects involved in the study according to the Declaration of Helsinki. In order to verify whether participants had the capacity to consent, we adopted the mini-mental state examination [43,44]. Specifically, all enrolled participants were characterized by a score greater than 24.

In the Neurorehabilitative Unit of Cisanello Hospital (Pisa, Italy), the analysis of muscle activation in post-stroke patients is routinely performed on a daily basis during the neuro-rehabilitative treatment for the recovering of walking capabilities, as part of institutional activities. Therefore, it is implicitly approved by the Ethical Committee review board of Cisanello Hospital (Pisa, Italy).

### Settings

All participants were asked to walk on a treadmill without body weight support and handrail. EMG signals of 12 ipsilateral leg muscles belonging to both limbs (Peroneus Longus, PERL, Gastrocnemius Lateralis, GL, Soleus, SOL, Tibialis Anterior, TA, Rectus Femoris, RF, Vastus Medialis, VM, Biceps Femoris, BF, Semitendinosus, ST, Adductor Longus, ADD, Tensor Fascia Latae, TFL, Gluteus Maximus Gmax, Gluteus Medius, Gmed) were recorded (NORAXON, Telemyo 2400 T, V2) using bipolar Ag-AgCl surface EMG electrodes placed on previously shaved and cleaned skin. Sampling rate was 1500 Hz and the gain of the amplifiers was 1000. Electrode placement was tested through suitable movements [45] in order to verify the absence of cross-talk among muscle signals. Subjects donned shoes provided with foot switches to record the heel strike event from both feet.

### Protocol

All participants were asked to walk on a treadmill at four controlled speeds: 0.5, 0.7, 0.9, and 1.1 km/h. The maximum speed was established to allow a comfortable pace on the treadmill without aids and handrail to all patients. Before starting EMG records, all subjects practiced treadmill locomotion for three minutes in accordance with previous literature [46,47]. For a given speed, each experimental session was long enough to account for at least 10 consecutive and bilateral gait cycles. Subjects carried out walking tests at different speeds in random sequence. All participants could stop sessions and ask for a resting period. In this case, pauses of at least five minutes were provided and, if required, the recording session was repeated.

### Spatio-temporal parameters

In order to estimate the walking performance and to assess the asymmetry, spatio-temporal parameters were obtained by analyzing heel strike events while subjects walked on the treadmill. The estimated parameters were **cadence** (steps/min) and **stride length** (m) for the healthy and the pathologic subjects, and **step duration** (s) of both affected and unaffected limbs and healthy subjects.

### EMG analysis and extraction of muscle synergies

EMG signals were pre-processed in accordance with previous literature [18]. Briefly, raw EMG signals were full wave rectified, low-pass filtered with a zero-lag Butterworth filter (4^th^ order, cut-off frequency at 10 Hz), and separated with respect to the gait cycles. These were then time-interpolated over 200 points and averaged across all strides in order to have a representative data set for each leg. Data related to healthy people were averaged across legs in order to obtain a single representative data set for each subject. This preliminary procedure provided a 200×12 matrix, which was later manipulated in order to extract muscle synergies and to estimate the spatio-temporal MN activity related to the rostro-caudal segments of the spinal cord. The mean value of the pre-processed EMG signals (**EMG**_**m**_) across the gait cycle was adopted to describe the amplitude of myoelectrical signals in both groups.

For each data set, Bartlett's test of sphericity and Kaiser-Meyer-Olkin (KMO) measure were computed in order to verify whether matrices were adequate for factorization. Then, each dataset was factorized using the Factor Analysis of the correlation matrix with varimax rotation (FA) in order to extract primitive signals, representing the timing of activation, and weight coefficients, representing muscle enrolment, related to each synergy. The number of retained synergies was identified by using the criterion of the *eigenvalue > 1* [1,18,48]. According to this criterion, the factors associated to the last eigenvalues (i.e., *eigenvalue < 1*) only account for a small percentage of the whole data variance and can be excluded. Finally, extracted synergies were pooled in homologous groups across subjects after estimating the best matching scalar product of the unit vectors obtained by dividing the weight coefficient vectors to their own Euclidean norms.

### Consistence of muscle synergies

As previously described, the EMG factorization is an algebraic process which basically consists in computing a *m* × *p* weight coefficient matrix that linearly projects the *p-*variables dataset of EMG signals (*p = 12* in this case), onto a *m-*dimensional one, i.e., the primitive signals.

The outcome of this process depends on two main choices: the method adopted to factorize the dataset and the criteria used to retain meaningful factors. With respect to the former issue, previous authors have already demonstrated that the FA is one among the best performing algorithms to identify correct muscle synergies [49,50]. Concerning the identification of meaningful factors, Ivanenko and colleagues [1] showed that the criterion of the *eigenvalue > 1* could result too stringent and would have not allowed the authors to extract some basic activation patterns underlying EMG signals of leg muscles while walking. Accordingly, they adopted the criterion of the *eigenvalue > 0.5* and, consequently, retained five components. This assumption was tested but not confirmed in one of our previous studies dealing with the same topic investigated by Ivanenko and colleagues [18]. In particular, we showed that three over five retained factors, i.e., those extracted by using the criteria of *eigenvalue > 1*, actually reflected the elementary biomechanical demands during the gait cycle while the remaining ones (i.e., *eigenvalue < 1*) did not provide any distinctive and univocal information. We hence concluded that the synergies extracted in accordance with the criteria of *eigenvalue > 1* were the most informative ones.

In order to verify whether retained modules could be considered meaningful, we compared weight coefficients and primitive signals when adopting both the criterion of *eigenvalue > 1* and the criterion of *eigenvalue > 0.5*. In this respect, the scalar product and the Pearson’s correlation coefficient were the metrics used to compare respectively the weight coefficients and the primitive signals between the two criteria.

Finally, since the aim of this study consists in comparing muscle synergies related to two different groups of participants, we focused our attention on the solution revealing meaningful common features across all subjects.

### Estimation of spatiotemporal MN activity

Pre-processed 200x12 matrices of EMG signals were used to estimate the spatiotemporal MN activity along the rostrocaudal direction related to the S2-L2, as described in literature [18,31,32,35]. Briefly, for each spinal segment, the indirect measure of MN activity along the gait cycle was computed as the weighted summation of all EMG signals innervated by such segment, where weight coefficients are those tabled in literature [32].

The main assumption underlying the estimation of the spinal maps is that EMG signals, after full-wave rectification and filtering, can provide an indirect measure of the net MN firing rate. This hypothesis has been assumed in accordance with earlier studies showing that EMG activity increases fairly linearly with the net motor unit firing rate both in young and in elderly people [51-53].

Then, the Center of Activity (**CoA**) was also computed in accordance with the equation reported by Ivanenko and colleagues [32], corresponding to the centroid of activity across all spinal segments for each time point. The **CoA** represents a synthetic measure of the weighted level of the spinal activity. For instance, a low **CoA** indicates a predominant involvement of muscles innervated by the caudal segments, while a high **CoA** indicates the prevalent activation of the muscles innervated by the rostral segments.

### Metrics for analyzing muscle synergies and spinal maps

The similarity between two sets of homologous muscle synergies was estimated by:

- the scalar product between weight coefficients vectors normalized to their own norms (**dot)**;
- the Pearson's correlation coefficient between their related primitive signals (**ρ**).

Metrics adopted to describe the similarity between two different spinal maps were:

- the 2D Pearson’s correlation coefficient between the two maps (**ρ**_**2D**_);
- the Pearson's correlation coefficient (**ρ**_**CoA**_) and the mean distance (**d**_**CoA**_) of their related **CoA**s.

For each metric (i.e., **dot**, **ρ**, **ρ**_**2D**_, **ρ**_**CoA**_, and **d**_**CoA**_), the averaged value obtained by comparing each patient, either affected or unaffected legs, and all healthy subjects was considered representative of the degree of similarity between that patient and the healthy control group. Then, results were pooled with respect to the walking speed and the observed limb (i.e., affected or unaffected), and compared by means of statistical tests.

### Statistics

In this study, the ANalysis Of Variance (ANOVA) for repeated measures was the main statistical test to investigate the effect of factors "speed" (i.e., 0.5, 0.7, 0.9, and 1.1 km/h), "side" (i.e., affected, unaffected, and healthy limbs), and “group” (i.e., healthy subjects and post-stroke survivors) on data variability.

More in detail, the two-way ANOVA for repeated measures was used to verify which among factors "speed" and "side" influenced data variability of **step duration**, **EMG**_**m**_**,** and all metrics describing muscle synergies and spinal maps (i.e., **dot**, **ρ**, **ρ**_**2D**_, **ρ**_**CoA**_, and **d**_**CoA**_). Noticeably, with respect to the factor “side”, datasets related to healthy limbs and affected/unaffected limbs were pooled in two separate sessions, once to compare the affected side of post-stroke patients to the healthy limbs, and again to compare the unaffected side of post-stroke patients to the healthy limbs, in accordance with previous literature [54,55].

The two-way ANOVA for repeated measures was also used to verify which among factors "speed" and "group" influenced the spatio-temporal parameters (i.e., **cadence** and **stride length**).

Finally, the anthropometrical features (age, weight, and height) of the healthy and post-stroke participants were compared by using a Wilcoxon signed-rank test, in order to test the homogeneity of the control group.

Significance was set at α = 0.05. Data analysis was implemented by using custom written MATLAB (The MathWorks, Natick, MA) scripts.

## Results

### Participants

Most of the patients (Table 1) were old adults (58-78 years old) and experienced a stroke more than 27 months before the experimental sessions (range: 27-63 months). Only two chronic patients (P2 and P12) were young adults (respectively 33 and 23 years old), and only two patients (P7 and P10) reported a cerebrovascular accident within the last year (respectively 2 and 8 months) before the experimental sessions. About half of patients needed a support, that is, cane or crutch, during daily activities.

Healthy participants were two young (respectively 20 and 35 years old) and eight old adults (age: 63.3 ± 3.1 years), and were characterized by anthropometric features comparable to those of post-stroke patients (weight: 76.6 ± 13.8 kg; height: 172.4 ± 6.5 cm, p > 0.60 for age, weight and height).

### Spatio-temporal parameters

**Figure 1:**
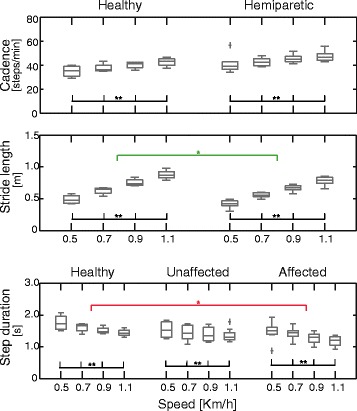
Spatio-temporal parameters. Boxplots concerning measured spatio-temporal parameters at four walking speeds for both healthy and hemiparetic subjects. The significance of the difference between groups (green lines for the comparison healthy-hemiparetic subjects, and red lines for the comparison healthy-affected limb) or among speeds (black lines) is indicated as follows: * p < 0.05; ** p < 0.01.

The factor “speed” had a significant (p < 0.001) effect on **cadence**, **stride length** and **step duration**, for healthy subjects and post-stroke patients. In particular, when the speed increased, the first two parameters increased and the last one decreased (Figure 1).

The **stride length** of post-stroke patients was significantly (p = 0.033) smaller than that of the healthy subjects (Figure 1). This result was accompanied by the evidence that the **cadence** of post-stroke patients was slightly even though not significantly faster than that of the control group (p = 0.058). The **step duration** of only the affected side was significantly (p = 0.048) smaller than that of healthy subjects.

### EMG signals

**Figure 2:**
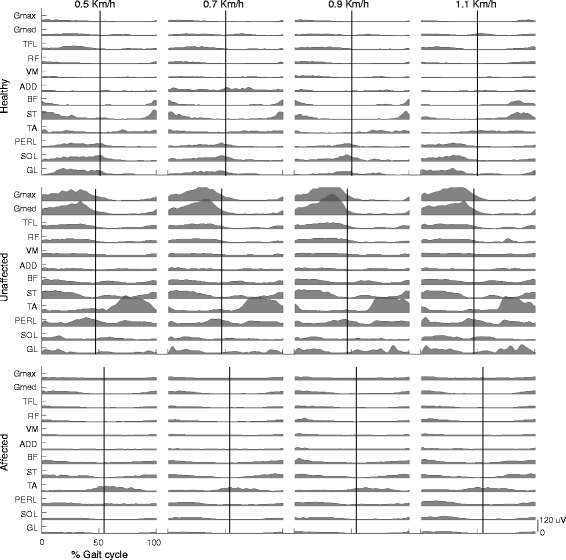
EMG signals. Representative sets of pre-processed EMG signals related to the twelve lower limb muscles of a healthy and a hemiparetic subject (P1) while they walked at the four walking speeds. The black vertical lines indicate the contralateral heelstrike (individual mean across gait cycles).

EMG signals of healthy subjects (Figure 2) were comparable to those reported in literature referring to the same speed [1,56,57].

**Figure 3:**
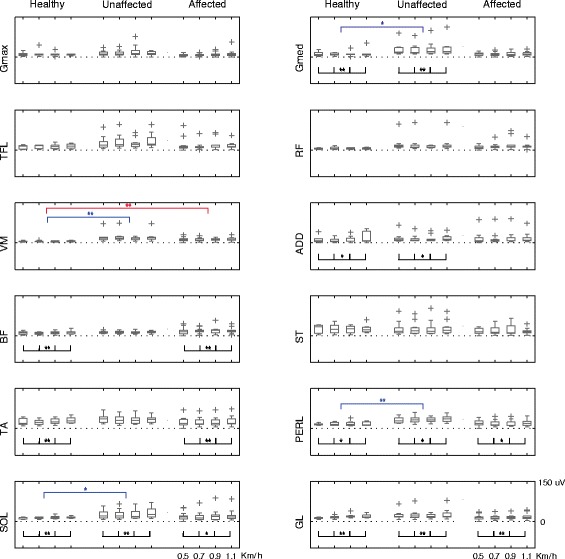
Mean activity of EMG signals. For each muscle, the boxplots represent intra-group mean EMG activity for the healthy, unaffected and affected limbs at the four speeds. The significance of the difference between groups (blue lines for the comparison healthy-unaffected limb and red lines for the comparison healthy-affected limb) or among speeds (black lines) is indicated as follows: * p < 0.05; ** p < 0.01.

Also EMG signals of post-stroke patients were similar to what previously shown [22,23,58-60] and, as expected, appeared altered respect to the ones of the control group, revealing that the myoelectric activity of the unaffected side was generally higher than that of the control group (Figure 3). This trend was evident in almost all muscles even though it was statistically significant (p < 0.05) in Gmed, VM, PERL and SOL. With respect to the affected side, only the rectified amplitude of VM was characterized by a mean value greater than that of the healthy subjects while the other muscles assumed comparable values between the groups.

Noticeably, in both sides of post-stroke patients, the TA showed a very low activity during the early stance, whereas it was mainly active during the swing phase. Furthermore, calf muscles, particularly in the unaffected limb, showed early and continuous activation during the stance phase. At all speeds, knee flexor and extensor muscles usually appeared co-activated.

As expected, the amplitude of EMG signals increased with speed, even though this trend was statistically significant (p < 0.05) only in muscle groups crossing ankle and hip (Figure 3).

### Muscle synergies

#### Consistence of muscle synergies

**Figure 4:**
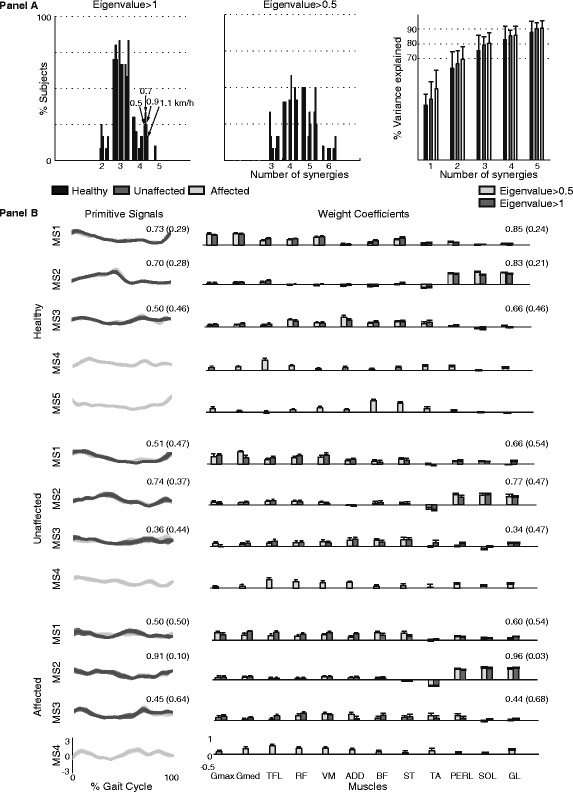
Muscle synergies: comparison between the retaining criteria. **Panel A**. On the top left, the percentage of subjects presenting different number of synergies, according to the criteria of the *eigenvalue > 1* and the *eigenvalue > 0.5*, is reported. For each limb (healthy in black, unaffected in dark gray, affected in light gray), bars represent the result achieved for all ordered walking speeds (i.e., 0.5, 0.7, 0.9, and 1.1 km/h). On the top right, the explained cumulative variance (mean ± standard deviation errorbar) of five retained synergies is reported. The explained cumulative variance refers to the fastest walking speed. The **Panel B** shows primitive signals (on the left) and weight coefficients for homologous muscle synergies retained according to both criteria (the dark gray is for the *eigenvalue > 1* and the light gray is for the *eigenvalue > 0.5*), and related to the fastest pace. Mean (standard deviation) of the Pearson correlation coefficients between homologous temporal components and mean (standard deviation) of the normalized scalar products between weight coefficients are reported on both features of muscle synergies.

Figure 4 shows the number of modules extracted by using both the criteria of *eigenvalue > 1* and *eigenvalue > 0.5* across all subjects and all speeds (panel A), and the features of muscle synergies (i.e., explained variance, weight coefficients and primitive signals; panel B) obtained with the two criteria across all subjects at 1.1 km/h. Based on the former criterion, almost all datasets could be decomposed by using three meaningful muscle synergies accounting, on average, for more than the 75% of data informativeness. Noticeably, the number of meaningful synergies was not affected by the pace. Differently, the latter one allowed us to generally retain between four and five muscle synergies, highlighting a wider inter-subjects and inter-speeds variability and accounting for more than 80%, in case of 4 synergies, and 85%, in case of 5 synergies, of data informativeness. According to the criterion of *eigenvalue > 0.5,* five muscle synergies can be considered meaningful for the healthy subjects and four muscle synergies for the affected and unaffected side of the post-stroke subjects.

Comparing weight coefficients and primitive signals (Figure 4, panel B), we observed that the first two modules retained in accordance with the criterion of *eigenvalue > 1* were very similar to the homologous ones retained according to the criterion of *eigenvalue > 0.5* (for all groups, the Pearson’s correlation coefficients between primitive signals ranged between 0.50 and 0.90 while the scalar product between homologous normalized weight coefficients ranged between 0.60 and 0.96). The third module showed lower similarity in both primitive signals and muscle synergies when comparing the two criteria (the Pearson’s correlation coefficient between primitive signals ranged between 0.36 and 0.50 while the scalar product between homologous normalized weight coefficients ranged between 0.34 and 0.66).

#### Analysis of retained muscle synergies

**Figure 5:**
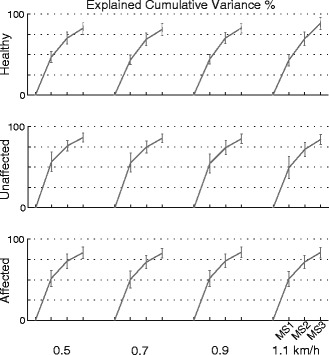
Cumulative variance of data sets. Explained cumulative variance accounted by extracted synergies in healthy, unaffected and affected limbs. Mean and standard deviations (error bars) are represented. **MS1**, **MS2** and **MS3** refer to the retained synergies.

Pre-processed data for both healthy and hemiparetic subjects were adequate for factorization (sphericity p < 0.001; KMO > 0.60). In all cases, according to the criterion of *eigenvalue > 1*, we retained three synergies to suitably describe data variability of each data set which accounted for more than 75% of the cumulative variance of data sets (Figure 5). Results also showed that in post-stroke patients the first synergy explained a variance greater than in healthy subjects, and this behavior was mainly emphasized in the unaffected limb (Figures 4 and 5).

**Figure 6:**
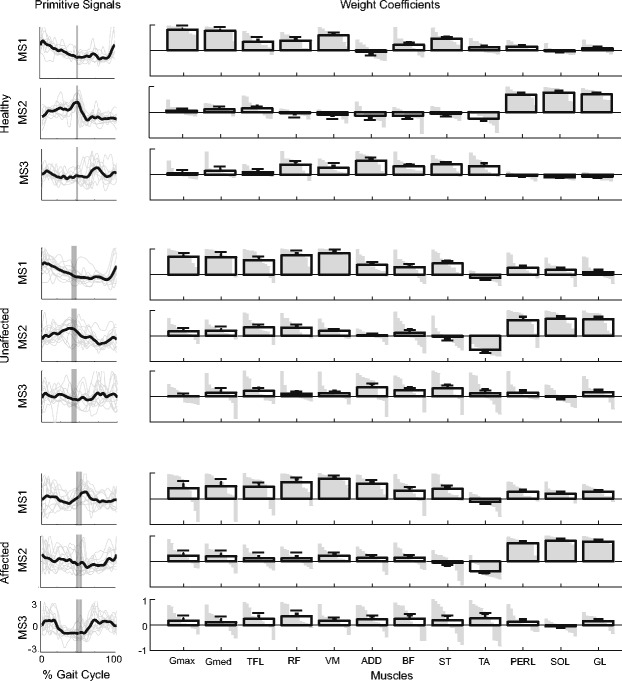
Muscle synergies. Primitive signals and weight coefficients related to extracted muscle synergies (**MS1**-**MS3**) according to the criterion of the *eigenvalue >* 1 and referring to all healthy and hemiparetic subjects while walking at 1.1 km/h. The averaged temporal components and the averaged weight coefficients are reported in black. Error bars refer to the standard error. White vertical lines and gray vertical bars overlapped to the primitive signals refer respectively to mean and one standard deviation of the occurrence of the contralateral heel strike.

The three muscle synergies extracted from healthy subjects (Figure 6) were comparable to those reported in literature [1,10-12,18]. Briefly:

- the first synergy (**MS1**) functionally referred to body support during weight acceptance, reflecting the activity of hip and knee extensors (e.g., Gmax, ST, BF, VM, RF), Gmed, TFL; its primitive signal showed a burst during the early stance (~10% of the gait cycle);
- the second synergy (**MS2**) mainly accounted for the activity of calf muscles referring to body support, forward propulsion, and swing initiation; its primitive signal was characterized by a peak at about 50% of the gait cycle;
- the third synergy (**MS3**) described the concomitant activation of knee extensor muscles (e.g., VM and RF), biarticular hip extensors (e.g., BF and ST), ADD and TA; its primitive was characterized by a main peak at 75% of the gait cycle.

In general, muscle synergies of post-stroke patients (Figure 6), in both affected and unaffected sides, were characterized by weight coefficients comparable to those of healthy subjects. Moreover, primitive signals of post-stroke subjects showed a variable shape compared to those of the healthy controls, especially in the affected limb. The third synergy appeared less consistent across subjects, and was loaded by several spread muscle groups.

**Figure 7:**
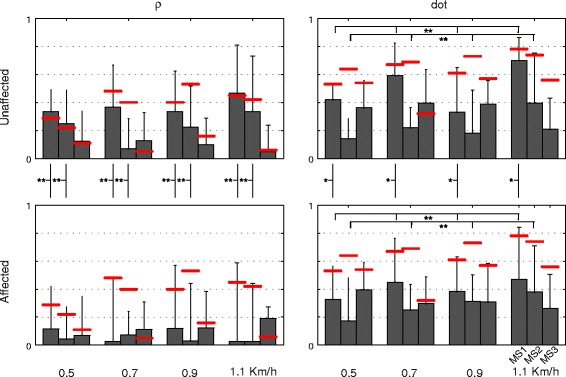
Metrics for muscle synergies comparison. Mean and standard deviation (one side error bar) of the metrics (i.e., **dot**, **ρ**) adopted to describe the similarity between muscle synergies of healthy and hemiparetic patients at each walking speed. The three bars of each walking condition are respectively referred to **MS1**, **MS2** and **MS3**. The red lines are the intra-group degree of similarity, in terms of **dot** and **ρ,** related to the healthy control group. The significance of the difference between groups (black vertical lines for the comparison unaffected-affected limb) or among speeds (black horizontal lines) is indicated as follows: * p < 0.05; ** p < 0.01.

The two-way ANOVA highlighted that the speed significantly affected weight coefficients of **MS1** (p = 0.003) and **MS2** (p = 0.027) in the affected and unaffected side, showing that when the speed increased, **dot** increased too (Figure 7). Concerning the difference between the sides (i.e., affected vs unaffected), the statistical analysis revealed a significant effect on both **MS1** and **MS2**. In particular, **ρ** related to **MS1** and **MS2** (p < 0.001 for **MS1** and p = 0.001 for **MS2**), and **dot** related to **MS1** (p = 0.042) were significantly higher in the unaffected side than in the contralateral one. Moreover, the intra-group similarity of healthy subjects was greater than that between healthy and hemiparetic patients (Figure 7).

### Spinal maps

**Figure 8:**
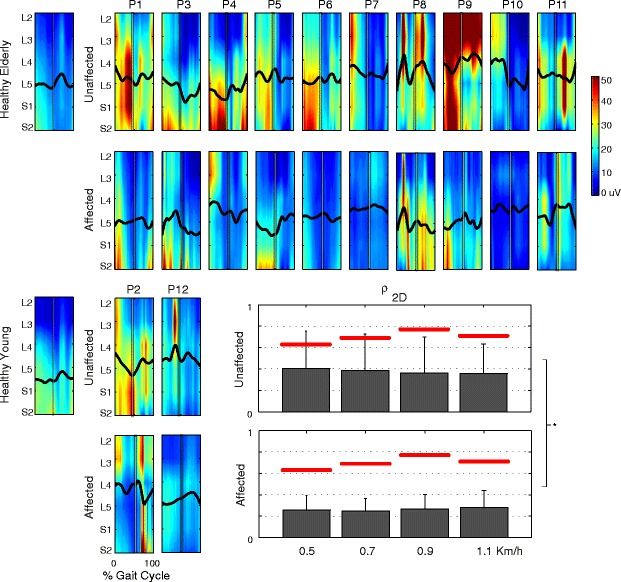
Estimated motoneuronal activity in the lumbosacral enlargement. The figure shows the spinal maps of all patients (elderly and young related patients respectively on the top and the bottom of the figure), those referring to the ensemble average across young and elderly healthy subjects (on the left of the panel), and their related **CoA**s (black lines). All spinal maps refer to the fastest walking speed. The white vertical lines and the gray bars overlapped to each spinal map indicate mean and standard errors of the onset of the contralateral heel strike. On the bottom, mean and standard deviation (one side error bar) of the **ρ**_**2D**_ related to the similarity between unaffected and affected side of post-stroke patients and healthy subject values at all speeds are reported. The red lines reflect the intra-group degree of similarity, in term of **ρ**_**2D**_, between spinal maps of the healthy control group. The significance of difference between sides (black line) is indicated as * p < 0.05.

The spinal maps of (young and elderly) healthy subjects (Figure 8) were similar to those reported in literature at comparable speed [13,18,32]. Young subjects maps were mainly characterized by four activation periods centered at about 10%, 45%, 75% and 95% of the gait cycle, primarily located in caudal segments (L4-S2), corresponding to the main phases of the gait cycle. In elders, MN activity was more spread involving one main burst during the stance phase and two in the activation phases at 75% and 95% [18].

The spinal maps of post-stroke participants highly differed from those of the control group, and were characterized by a wide intra-group variability (Figure 8). Moreover, they were qualitatively dissimilar between affected and unaffected limbs.

The MN activity referring to the unaffected limb was generally more intense and extended than that related to the affected side (Figure 8). It was characterized by intense bursts during the stance phase spread along the whole rostro-caudal enlargement (see P1, P5, P7, P9, P11, and P2 in Figure 8), or it showed a main burst in the rostral (see P8, P10, and P12 in Figure 8) or in the caudal sections (see P3, P4, and P6 in Figure 8). During the swing phase, the MN patterning across patients showed greater variability, involving all segments of the rostro-caudal enlargement (Figure 8).

Spinal maps related to the affected side were generally characterized by lower amplitude than those referring to the contralateral side (Figure 8). They presented an intense activity during the stance phase in most caudal segments (see P1, P3, P5, P8, and P9 in Figure 8), or bursts along more rostral ones (see P4, P1 and P2). Moreover, four patients (P6, P7, P10 and P12) were characterized by very low activation of the spinal circuits in the whole gait cycle.

According to previous works [32,36], the **CoAs** of both healthy and hemiparetic subjects showed periodical rostral-caudal oscillations during the gait cycle (Figure 8).

**Figure 9:**
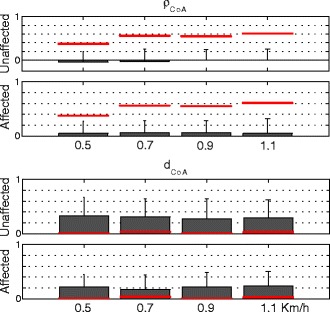
Metrics describing the CoAs. Mean and standard deviation (one side error bar) of metrics adopted to describe the similarity between **CoA**s of healthy and the affected and unaffected side of hemiparetic patients. The red lines on the upper subplots reflect the intra-group degree of similarity, in term of **ρ**_**CoA**_ between spinal maps, of the healthy control group. Those on the lower subplots represent the averaged **d**_**CoA**_ estimated for only healthy subjects.

The analysis of the spinal maps showed that the spatio-temporal MN activity of the unaffected limb was significantly (p = 0.004) more similar to that of healthy subjects than the contralateral side (see **ρ**_**2D**_ in Figure 8). Moreover, the **CoA** related to unaffected limb was shifted toward more rostral segments (Figure 9) compared to the affected limb, even though the difference was not statistically significant (p = 0.072). The **ρ**_**CoA**_ was always close to zero and did not show significant trend between the sides. Finally, the walking speed did not influence metrics describing spinal maps (i.e., **ρ**_**2D**_, **ρ**_**CoA**_, and **d**_**CoA,**_ p > 0.05).

It is important to remark that all metrics describing the degree of similarity of spinal maps between healthy subjects and post-stroke patients (i.e., **ρ**_**2D**_, **ρ**_**CoA**_, and **d**_**CoA**_) were always lower than the intra-group level of similarity of the healthy control group.

## Discussion

This study was designed to investigate the effects of a unilateral cerebrovascular accident on muscle synergies and spinal maps underlying the activation of leg muscles while walking. In particular, it was aimed at clarifying whether the asymmetric gait of post-stroke survivors can be related to the unbalanced recruitments of motor modules underlying the activity of leg muscles between the two limbs, as described by muscle synergies, and/or to the altered spatio-temporal organization of the output of the CPG, as described by spinal maps.

### Consistence of muscle synergies

As first result, our study confirms that the activity of twelve leg muscles in healthy and post-stroke subjects while walking can be expressed as the summation of a limited number of suitably weighted primitive signals [24,26,34]. With respect to the number of retained synergies, our analysis revealed that the criterion of *eigenvalue > 1* allowed us identifying a set of modules common across groups and walking speeds and referring to the elementary biomechanical demands during the gait cycle (i.e., loading response, push-off and heel strike preparation). Conversely, as expected, the criterion of *eigenvalue > 0.5* allowed us to identify more modules with a greater explained variance even if they were not characterized by unequivocal and distinctive information across subjects and speeds.

Indeed, from the algebraic viewpoint, the number of retained synergies has been defined as the threshold between capturing systematic behavior and random fluctuations [61] within a dataset which is itself greatly variable [57]. Unfortunately, when translated in practical criteria to disentangle the signal from noise, this statement does not provide unequivocal conclusions. As matter of facts, previous authors [1,2,10-12,24,26,32,36], adopting several criteria (e.g., eigenvalue > 1, maximum cumulative variance, "a posteriori" analysis of muscle synergies, VAF estimated across six different portions of the gait cycle), have extracted from two to five synergies underlying muscle activity related to 7-30 both ipsilateral and bilateral muscles. This discrepancy could be ascribed to the absence of agreement concerning the pre-processing algorithms [62], to the identification of suitable criteria to extract the significant communality [29], and to the different set of muscles included in the analysis [63]. Moreover, the wide variability of the features of the pathology (e.g., the severity of the trauma, the time elapsed from the onset, the location of the accident, etc.) could have further exacerbated the discrepancies among the authors [24,26]. According to these evidences, how to select the most informative set of muscle synergies is still an open issue which requires further investigations to be unequivocally answered.

With respect to this study, three muscle synergies were consistently considered significant to reconstruct muscle activity in both healthy subjects and post-stroke patients, based on the assumption that a factor can be considered significant if its explained variance is at least as much as that of one original variable (i.e., criteria of the *eigenvalue > 1*, Figures 4 and 5) [10,18,48]. This outcome was further corroborated by the evidence that each of the retained synergies could be interpreted in terms of known biomechanical demands whereas the remaining ones did not show unequivocal and distinctive information, in accordance with the guidelines reported by Ting and Chvatal. [9]. Accordingly, since the purpose of our study consisted in comparing muscle synergies between post-stroke patients and healthy subjects, as done in previous studies [28,29], we focused our attention only on the first three modules.

### Muscle synergies

Muscle synergies extracted in both groups (Figure 6) were qualitatively in agreement with those reported by previous authors [18,24,26], reflecting a modular organization of the biomechanical demands during locomotion (i.e., loading response, push-off, lowering the swinging leg before the heel strike). With respect to the laterality, **MS1** and **MS2** appeared significantly sensitive to the observed side (i.e., affected and unaffected; see Figure 6 and 7), such that primitive signals and weight coefficients of the unaffected side resulted more similar to that of healthy subjects than those related to the contralateral one. Accordingly, this result readily suggested that muscle coordination underlying the unaffected limb was more comparable to that of healthy subjects than the contralateral one.

Actually, the asymmetry of muscle synergies has not been unequivocally observed in literature. In particular, some authors, [8,26] reported similar modular organization underlying muscle activity, apart from observed side, size and location of the lesion among patients. Others [24] were instead able to discriminate between the sides, due to the number of modules, which independently characterized muscle activation of affected and unaffected limbs (i.e., the number of synergies was lower for the affected limb). In our population of patients, we also observed that the variance explained by the components related to the affected side was generally higher than that related to the unaffected side, and both of them were generally higher than that of healthy modules (Figures 4 and 5). Nonetheless this difference was not enough significant to discriminate between subjects and limbs.

In our opinion, the attitude to distinguish between affected and unaffected limbs due to both the reduced algebraic complexity of data sets [24], or their similarity with those of healthy subjects (as in this study), reflects the same features. In particular, a cerebrovascular accident involves a significant coactivation of muscles crossing proximal leg joints, and induces abnormal temporal patterning above all in the affected limb [23,58,59]. In terms of factorization, this means that agonist and antagonist muscle groups present greater correlation, promoting their ensemble grouping above all in the affected limb. On the other hand, their irregular temporal patterning is reflected in abnormal muscle activities, allowing muscle synergies of affected and unaffected limbs to be distinguished, as in this study.

Another interesting result was that the difference between affected and unaffected limbs was significantly relevant only for the first two synergies, and was not observed in **MS3**. From one hand, this allows hypothesizing that the poor motor control due to the stroke does not significantly modify the lowering of the leg before achieving the heel strike (i.e., functional representation of **MS3**). Moreover, due to the slow speeds, the lowering of the leg can result in a quite ballistic control such that the contribution of posterior thigh musculature and the TA on **MS3** was negligible. On the other hand, since **MS3** accounted for the lowest percentage of data information (Figure 4), its intrinsic variability could not allow highlighting any systematic behavior limiting the possibility to distinguish between the two sides.

### Spinal maps

The first expected result concerning spinal maps was that their activity is reorganized after a cerebrovascular accident, involving great variability of timing, amplitude, and distribution of bursts between affected and unaffected limbs and across patients (Figure 7). Moreover, the spinal maps estimated for the unaffected side were more similar to the maps of the healthy group than those referring to the contralateral side (Figure 8).

Despite the wide inter-subjects variability, the **d**_**CoA**_ (Figure 9) appeared slightly influenced by the observed side (p = 0.072), highlighting that the **CoA** related to the unaffected limb was shifted to more rostral segments than that related to the contralateral side. This suggests a greater workload of proximal muscle groups in the unaffected leg. Actually, previous authors already observed that the significant coactivity of muscles crossing hip and knee could have a functional relevance in post-stroke patients [23,59,60]. Moreover, the significant contribution of proximal muscle groups was also shown to reflect neuromuscular adaptation due to ageing, neurological disorders, and/or presence of comorbidities [47,64]. Although a stroke is expected to elicit compensative and abnormal strategies in the unaffected limb aimed at increasing stability and efficiency of locomotion, spinal maps highlight that these strategies are achieved by the involvement of proximal muscle groups in the unaffected limb. In other words, the estimated spatio-temporal activity of MN pools can provide a synthetic representation of the asymmetry in muscle coordination induced by a unilateral cerebrovascular accident. It is shown by the plastic redistribution of the burst of activity.

### Possible origins of the asymmetric gait after stroke

The results of this work showed that muscle synergies and spinal maps related to the unaffected side result more similar to the ones of healthy subjects than those related to the affected side, reflecting the asymmetry in muscle coordination between sides underlying the asymmetric gait after stroke. Noticeably, although we tried to quantify, ex post, the correlations between the asymmetry in gait patterns and that related to muscle synergies and spinal maps (data not reported), results were not consistent across conditions possibly due to the reduced sample size.

From the physiological viewpoint, the results suggest that the primitive signals activating muscle synergies can be significantly influenced by modifications of higher levels of the CNS, such as those due to a stroke. This leads to speculate that the trauma affects signals coming from higher levels of the CNS, such as premotor circuits, which enable/modulate the coordinated activity of neuronal networks encrypting muscle synergies [27]. However, it is relevant to observe that the expected involvement of higher regions of the CNS while eliciting muscle synergies has not been unanimously reported among authors [8,24,26,27] probably due to their different methodological approaches.

Indeed, we believe that the altered primitive signals underlying muscle synergies related to the affected side may reflect both the modifications of higher levels of the CNS resulting after the trauma and the altered sensory-motor feedback, as shown by the spinal maps. Specifically, since the activation of muscle synergies is somehow modulated by the afferencies [2], the altered gait patterns of post stroke patients might provide abnormal feedback to the patter generator which can affect the primitive signals and, consequently, may also abnormally elicit the muscle recruitment in muscle synergies. As matter of the fact, it has been recently demonstrated that when healthy and post-stroke subjects are asked to walk with the same dynamical conditions imposed by a fixed speed, the accident mainly affects the intralimb coordination patterns suggesting that the hemiparetic gait can be achieved by adopting a wide range of possible coordination patterns underlying specific optimization roles [54]. The high pathology-related inter-subjects variability is hence expected to influence the afferent feedback to the CNS and, therefore, to modulate the primitive signals activating muscle synergies.

This hypothesis is also in agreement with the recent finding in the mouse spinal cord that the motor synergy encoder, i.e. population of neurons in the spinal cords with key features related to motor synergies, receives inputs from sensory pathways and from pyramidal cells in the motor cortex [65].

On the whole, although we hypothesized that the stroke-related alteration of muscle synergies may be due to the concomitant contributions of altered information from both the higher level of the CNS and the periphery, further and deep analysis of the coordinated activity of muscles while walking based on standardized and well-defined data processing, is still required.

### Limits of the study

We have to acknowledge that the main limits of this study are the reduced number of participants and the great inter-subjects variability in term of age, severity of the pathology, and elapsed time from the trauma. Further investigations on a larger cohort of post-stroke subjects have to be performed in order to confirm our results. Nevertheless, with respect to the aim of this study (i.e., to verify whether muscle synergies and spinal maps can be sensitive to the asymmetry of post-stroke patients while walking), the greater variability of data set could have kept the results of the statistical analysis more conservative. A further support for this conclusion results from the degree of inter and intra groups similarity. Specifically, although previous authors have already reported that muscle synergies and spinal maps are characterized by a certain variability across healthy subjects [1,13,18,32,66], and that the adopted slow speed could have introduced even more variability across them [1,18,56], all metrics (Figures 6, 7 and 8) showed that the degree of similarity between healthy and post-stroke patients was definitively lower than that representing the healthy intra-group one. This result reinforced the conclusion that muscle synergies and spinal maps are sensitive to the asymmetry resulting from the pathology. Therefore, noticed differences between affected and unaffected limbs reported in this study suggest that further significant discrepancies between sides could come up when a more homogeneous group of patients is observed.

Concerning the investigation of the origin of the asymmetric walking behavior in post-stroke subjects, the adopted experimental conditions (i.e., treadmill-based walking at controlled speed) may have impacted the results since it is well known that the speed, per se, affects in a significant fashion gait patterns [47,54]. Accordingly, we believe that our proposed analysis should be extended to more diversified motor tasks, such as walking on a treadmill and over ground at different speeds, to better prove that the muscle synergies and spinal maps are not task-dependent, but rather describe mechanisms underpinned at different neurophysiological levels.

Finally, we have to acknowledge that the segmentation in gait cycles was accomplished by using heel strike events, in accordance with previous authors [23,25,26,67,68]. Although this approach is not optimal to define the gait cycle in case of pathological subjects [69], we believe that it would not significantly affect the results since it would only and slightly influence the timing of the primitive signals, while the synergistic muscle coordination or the topography of estimated MN activity would not be influenced.

## Conclusion

On the whole, our results confirm that both muscle synergies and spinal maps reflect the asymmetry in EMG signals between affected and unaffected limbs of post-stroke patients while walking. In this regard, it is possible to speculate that the altered muscular organization after stroke as highlighted by muscle synergies and spinal maps may be due to the altered information coming from the upper part of the CNS, as resulting from the stroke, and/or to the abnormal sensory feedback due to the neuromuscular adaptation of the patients. However, further investigations are required to document the involvement of the upper levels of the CNS while carrying out motor tasks and to confirm that the coordinated activity underlying muscle recruitment may describe neuro-physiological mechanisms underlying the plasticity of the neuro-muscular system after stroke.

## Abbreviations

ADD: Adductor Longus
ANOVA: ANalysis Of Variance
BF: Biceps Femoris
CNS: Central Nervous System
CoA: Center of Activity
CPG: Central Pattern Generator
dot: the scalar product between weight coefficients vectors
EMG: Electromyographic
EMGm: mean value of the pre-processed EMG signals
FA: Factor Analysis
GL: Gastrocnemius Lateralis
Gmax: Gluteus Maximus
Gmed: Gluteus Medius
KMO: Kaiser-Meyer-Olkin measure
MN: Motoneuronal
NNMF: non-negative matrix factorization algorithm
PERL: Peroneus Longus
P1 to P12: Patient identification
RF: Rectus Femoris
SOL: Soleus
ST: Semitendinosus
MS1: MS2, MS3, muscle synergies
S2 to L2: rostrocaudal segments of the spinal cord
TA: Tibialis Anterior
TFL: Tensor Fascia Latae
VAF: Variability Accounted For
VM: Vastus Medialis
ρ: Pearson's correlation coefficient between timing activations
ρCoA: Pearson's correlation coefficient of the CoA of the spinal maps.
ρ2D: 2D Pearson’s correlation coefficient between the two maps

## References

[1] Ivanenko YP, Poppele RE, Lacquaniti F (2004). Five basic muscle activation patterns account for muscle activity during human locomotion. J Physiol.

[2] Ivanenko YP, Poppele RE, Lacquaniti F (2006). Motor control programs and walking. Neuroscientist.

[3] d'Avella A, Fernandez L, Portone A, Lacquaniti F (2008). Modulation of phasic and tonic muscle synergies with reaching direction and speed. J Neurophysiol.

[4] d'Avella A, Portone A, Fernandez L, Lacquaniti F (2006). Control of fast-reaching movements by muscle synergy combinations. J Neurosci.

[5] Bizzi E, Cheung VC (2013). The neural origin of muscle synergies. Front Comput Neurosci.

[6] D'avella A, Lacquaniti F (2013). Control of reaching movements by muscle synergy combinations. Front Comput Neurosci.

[7] Lacquaniti F, Ivanenko YP, D’Avella A, Zelik KE, Zago M (2013). Evolutionary and developmental modules. Front Comput Neurosci.

[8] Cheung VC, Piron L, Agostini M, Silvoni S, Turolla A, Bizzi E (2009). Stability of muscle synergies for voluntary actions after cortical stroke in humans. Proc Natl Acad Sci U S A.

[9] Ting LH, Chvatal SA, Danion F, Latash ML (2010). Decomposing muscle activity in motor tasks. Motor control theories, experiments and application.

[10] Davis BL, Vaughan CL (1993). Phasic behavior of Emg signals during gait - Use of multivariate-statistics. J Electromyogr Kines.

[11] Olree KS, Vaughan CL (1995). Fundamental patterns of bilateral muscle activity in human locomotion. Biol Cybern.

[12] Merkle LA, Layne CS, Bloomberg JJ, Zhang JJ (1998). Using factor analysis to identify neuromuscular synergies during treadmill walking. J Neurosci Methods.

[13] Cappellini G, Ivanenko YP, Poppele RE, Lacquaniti F (2006). Motor patterns in human walking and running. J Neurophysiol.

[14] Allen JL, Neptune RR (2012). Three-dimensional modular control of human walking. J Biomech.

[15] Neptune RR, Clark DJ, Kautz SA (2009). Modular control of human walking: a simulation study. J Biomech.

[16] Sartori M, Gizzi L, Lloyd DG, Farina D (2013). A musculoskeletal model of human locomotion driven by a low dimensional set of impulsive excitation primitives. Front Comput Neurosci.

[17] Patla AE (1985). Some characteristics of EMG patterns during locomotion: implications for the locomotor control process. J Mot Behav.

[18] Monaco V, Ghionzoli A, Micera S (2010). Age-related modifications of muscle synergies and spinal cord activity during locomotion. J Neurophysiol.

[19] Kargo WJ, Giszter SF (2008). Individual premotor drive pulses, not time-varying synergies, are the units of adjustment for limb trajectories constructed in spinal cord. J Neurosci.

[20] Huitema RB, Hof AL, Mulder T, Brouwer WH, Dekker R, Postema K (2004). Functional recovery of gait and joint kinematics after right hemispheric stroke. Arch Phys Med Rehabil.

[21] Knutsson E, Richards C (1979). Different types of disturbed motor control in gait of hemiparetic patients. Brain.

[22] Olney SJ, Richards C (1996). Hemiparetic gait following stroke. Part I: Characteristics. Gait Posture.

[23] Den Otter AR, Geurts AC, Mulder T, Duysens J (2006). Gait recovery is not associated with changes in the temporal patterning of muscle activity during treadmill walking in patients with post-stroke hemiparesis. Clin Neurophysiol.

[24] Clark DJ, Ting LH, Zajac FE, Neptune RR, Kautz SA (2010). Merging of healthy motor modules predicts reduced locomotor performance and muscle coordination complexity post-stroke. J Neurophysiol.

[25] Bowden MG, Clark DJ, Kautz SA (2010). Evaluation of abnormal synergy patterns poststroke: relationship of the Fugl-Meyer Assessment to hemiparetic locomotion. Neurorehabil Neural Repair.

[26] Gizzi L, Nielsen JF, Felici F, Ivanenko YP, Farina D (2011). Impulses of activation but not motor modules are preserved in the locomotion of subacute stroke patients. J Neurophysiol.

[27] Cheung VC, Turolla A, Agostini M, Silvoni S, Bennis C, Kasi P (2012). Muscle synergy patterns as physiological markers of motor cortical damage. Proc Natl Acad Sci U S A.

[28] Roh J, Rymer WZ, Perreault EJ, Yoo SB, Beer RF (2013). Alterations in upper limb muscle synergy structure in chronic stroke survivors. J Neurophysiol.

[29] Tropea P, Monaco V, Coscia M, Posteraro F, Micera S (2013). Effects of early and intensive neuro-rehabilitative treatment on muscle synergies in acute post-stroke patients: a pilot study. J Neuroeng Rehabil.

[30] Trumbower RD, Ravichandran VJ, Krutky MA, Perreault EJ (2010). Contributions of altered stretch reflex coordination to arm impairments following stroke. J Neurophysiol.

[31] Grasso R, Ivanenko YP, Zago M, Molinari M, Scivoletto G, Castellano V (2004). Distributed plasticity of locomotor pattern generators in spinal cord injured patients. Brain.

[32] Ivanenko YP, Poppele RE, Lacquaniti F (2006). Spinal cord maps of spatiotemporal alpha-motoneuron activation in humans walking at different speeds. J Neurophysiol.

[33] Yakovenko S, Mushahwar V, VanderHorst V, Holstege G, Prochazka A (2002). Spatiotemporal activation of lumbosacral motoneurons in the locomotor step cycle. J Neurophysiol.

[34] Ivanenko YP, Cappellini G, Dominici N, Poppele RE, Lacquaniti F (2007). Modular control of limb movements during human locomotion. J Neurosci.

[35] Ivanenko YP, Cappellini G, Poppele RE, Lacquaniti F (2008). Spatiotemporal organization of alpha-motoneuron activity in the human spinal cord during different gaits and gait transitions. Eur J Neurosci.

[36] Cappellini G, Ivanenko YP, Dominici N, Poppele RE, Lacquaniti F (2010). Migration of motor pool activity in the spinal cord reflects body mechanics in human locomotion. J Neurophysiol.

[37] Ivanenko YP, Grasso R, Zago M, Molinari M, Scivoletto G, Castellano V (2003). Temporal components of the motor patterns expressed by the human spinal cord reflect foot kinematics. J Neurophysiol.

[38] Ivanenko YP, Poppele RE, Lacquaniti F (2009). Distributed neural networks for controlling human locomotion Lessons from normal and SCI subjects. Brain Res Bull.

[39] Goulding M (2009). Circuits controlling vertebrate locomotion: moving in a new direction. Nature Rev Neurosci.

[40] Grillner S, Brooks VB (1981). Control of locomotion in bipeds, tetrapods, and fish. Handbook of physiology, section I: the nervous system, Vol. II: motor control, part 2.

[41] Fugl-Meyer AR, Jaasko L, Leyman I, Olsson S, Steglind S (1975). The post-stroke hemiplegic patient. 1. a method for evaluation of physical performance. Scand J Rehabil Med.

[42] Hauser SL, Dawson DM, Lehrich JR, Beal MF, Kevy SV, Propper RD (1983). Intensive immunosuppression in progressive multiple sclerosis: a randomized, three-arm study of high-dose intravenous cyclophosphamide, plasma exchange, and ACTH. New Engl J Med.

[43] Folstein MF, Folstein SE, McHugh PR (1975). “Mini-mental state”: a practical method for grading the cognitive state of patients for the clinician. J Psychiat Res.

[44] Tombaugh TN, McIntyre NJ (1992). The mini-mental state examination: a comprehensive review. J Am Geriatrics Soc.

[45] Hermens HJ, Freriks B, Disselhorst-Klug C, Rau G (2000). Development of recommendations for SEMG sensors and sensor placement procedures. J Electromyogr Kinesiol.

[46] Chen G, Patten C, Kothari DH, Zajac FE (2005). Gait differences between individuals with post-stroke hemiparesis and non-disabled controls at matched speeds. Gait Posture.

[47] Monaco V, Rinaldi LA, Macri G, Micera S (2009). During walking elders increase efforts at proximal joints and keep low kinetics at the ankle. Clin Biomech.

[48] Sabatini AM (2002). Identification of neuromuscular synergies in natural upper-arm movements. Biol Cybern.

[49] Ivanenko YP, Cappellini G, Dominici N, Poppele RE, Lacquaniti F (2005). Coordination of locomotion with voluntary movements in humans. J Neurosci.

[50] Tresch MC, Cheung VC, d'Avella A (2006). Matrix factorization algorithms for the identification of muscle synergies: evaluation on simulated and experimental data sets. J Neurophysiol.

[51] Day SJ, Hulliger M (2001). Experimental simulation of cat electromyogram: evidence for algebraic summation of motor-unit action-potential trains. J Neurophysiol.

[52] Zhou P, Rymer WZ (2004). Factors governing the form of the relation between muscle force and the EMG: A simulation study. J Neurophysiol.

[53] Masakado Y, Noda Y, Nagata MA, Kimura A, Chino N, Akaboshi K (1994). Macro-EMG and motor unit recruitment threshold: differences between the young and the aged. Neurosci Lett.

[54] Rinaldi LA, Monaco V (2013). Spatio-temporal parameters and intralimb coordination patterns describing hemiparetic locomotion at controlled speed. J Neuroeng Rehabil.

[55] Stephenson JL, De Serres SJ, Lamontagne A (2010). The effect of arm movements on the lower limb during gait after a stroke. Gait Posture.

[56] Shiavi R, Bugle HJ, Limbird T (1987). Electromyographic gait assessment, Part 1: Adult EMG profiles and walking speed. J Rehabil Res Dev.

[57] Winter DA (1991). The biomechanics and motor control of human gait : normal, elderly and pathological.

[58] Shiavi R, Bugle HJ, Limbird T (1987). Electromyographic gait assessment, Part 2: Preliminary assessment of hemiparetic synergy patterns. J Rehabil Res Dev.

[59] Den Otter AR, Geurts ACH, Mulder T, Duysens J (2007). Abnormalities in the temporal patterning of lower extremity muscle activity in hemiparetic gait. Gait Posture.

[60] Lamontagne A, Richards CL, Malouin F (2000). Coactivation during gait as an adaptive behavior after stroke. J Electromyogr Kinesiol.

[61] Cheung VC, d'Avella A, Tresch MC, Bizzi E (2005). Central and sensory contributions to the activation and organization of muscle synergies during natural motor behaviors. J Neurosci.

[62] Hug F, Turpin NA, Dorel S, Guevel A (2012). Smoothing of electromyographic signals can influence the number of extracted muscle synergies. Clin Neurophysiol.

[63] Steele KM, Tresch MC, Perreault EJ (2013). The number and choice of muscles impact the results of muscle synergy analyses. Front Comput Neurosci.

[64] McGibbon CA (2003). Toward a better understanding of gait changes with age and disablement: neuromuscular adaptation. Exerc Sport Sci Rev.

[65] Levine AJ, Hinckley CA, Hilde KL, Driscoll SP, Poon TH, Montgomery JM, Pfaff SL (2014). Identification of a cellular node for motor control pathways. Nat Neurosci.

[66] Cappellini G, Ivanenko YP, Dominici N, Poppele RE, Lacquaniti F (2010). Motor patterns during walking on a slippery walkway. J Neurophysiol.

[67] Kloter E, Wirz M, Dietz V (2011). Locomotion in stroke subjects: interactions between unaffected and affected sides. Brain.

[68] Kautz SA, Bowden MG, Clark DJ, Neptune RR (2011). Comparison of motor control deficits during treadmill and overground walking poststroke. Neurorehabil Neural Repair.

[69] Perry J, Davids JR (1992). Gait analysis: normal and pathological function. J Pediatr Orthoped.

